# Editorial: Probiotics and their metabolites in cancer therapy

**DOI:** 10.3389/fphar.2024.1497524

**Published:** 2024-10-09

**Authors:** Pourya Gholizadeh, Amir Hossein Faghfouri, Pio Maria Furneri, Elnaz Faghfuri

**Affiliations:** ^1^ Digestive Disease Research Center, Ardabil University of Medical Sciences, Ardabil, Iran; ^2^ Zoonoses Research Center, Ardabil University of Medical Science, Ardabil, Iran; ^3^ Maternal and Childhood Obesity Research Center, Urmia University of Medical Sciences, Urmia, Iran; ^4^ Department of Biomedical and Biotechnological Sciences (Biometec), University of Catania, Catania, Italy

**Keywords:** probiotics, cancer, gastrointestinal tract, digestion, gut microbiota

The bacterial strains of probiotics can be either naïve or genetically modified in any way. When utilized in dietary supplements, they are referred to as “live microbiological ingredients,” when used as a drug, they are known as “live biotherapeutic agents.” Most probiotics are categorized into bifidobacterial and lactic acid bacterial strains. However, certain bacteria and yeasts are also classified as probiotics. Probiotics play critical roles in preventing and effectively treating various types of cancer (Faghfuri and Gholizadeh, 2024; Feizi et al., 2024). Probiotics and their pro-bioactive cellular materials offer numerous therapeutic benefits for the gastrointestinal tract, including the release of various enzymes and potential synergistic effects on digestion. Specific components of probiotic lactic acid bacteria can modulate immune responses, activate the reticuloendothelial system, enhance cytokine pathways, and regulate interleukins and tumor necrosis factors. The primary mechanisms through which probiotics exert anticancer and antimutagenic effects include the binding, degradation, and inhibition of mutagens; preventing the production of procarcinogens and the transformation of harmful carcinogens; lowering the gut pH by producing short-chain fatty acids (SCFAs) from nondigestible carbohydrates; and enhancing the host’s innate immunity through the secretion of anti-inflammatory molecules. As essential dietary supplements, probiotics play a valuable role in reducing cancer risk and ensuring the safety of chemotherapy, radiation therapy, and surgery, with minimal side effects (Faghfuri and Gholizadeh, 2024; Feizi et al., 2024). Therefore, investigating potential probiotic strains, effective dosages, and molecular mechanisms that can support cancer therapy is crucial. This Research Topic highlights the potential roles of live and dead probiotics, along with their metabolic products, in cancer treatment and the exploration of alternative therapeutic mechanisms.

The Research Topic delves into five articles that thoroughly investigate the use of probiotics and their metabolites for treating various types of cancer. Recent studies have explored the potential of probiotics as oral vaccines, which carry fewer risks than pathogenic alternatives do. These oral vaccines can stimulate mucosal immunity, helping to protect against intestinal infections. Probiotic bacteria can produce metabolites such as anti-inflammatory cytokines, which are crucial for preventing cancer development and activating phagocytes to eradicate early-stage cancer cells. In addition to probiotics, their secreted products, including bacteriocins, exopolysaccharides, SCFAs, conjugated linoleic acid, peptidoglycan, and other metabolites, have shown anticancer properties. The beneficial effects of these postbiotic compounds have been extensively studied to understand their mechanisms of action in reducing cancer growth. Sudaarsan et al. focused primarily on the postbiotic components used against cancer and their documented mechanisms of action. This research also outlines recent studies conducted to examine specific strains and the anticancer activity of derived compounds, both in laboratory settings and in living organisms, confirming that the probiotic approach could offer an alternative strategy for alleviating the burden of cancer.

Singh et al. assessed the advantages and disadvantages of the use of oral probiotic vaccines and elucidated the mode of action of probiotics in treating colon cancer. Multiple studies have shown that healthy gut bacteria and probiotics can have significant effects on the immune system and metabolism of the host. Even a small disruption in the gut microbiome and its functions can lead to the development of colon cancer. They aid in cancer treatment, whether in the early stages of tumor development or during cancer therapy. The consumption of food rich in probiotics may lower the risk of colon cancer. Researchers are keen to explore various probiotic strains for their potential to induce cell death, presenting a promising strategy for managing colon cancer. The studies cited in this paper show that certain probiotic bacteria can influence cancer formation by producing cytokines and metabolites, regulating apoptotic genes, and reducing gut inflammation during cancer. However, the effectiveness of specific bacteria in preventing cancer can vary. Oral vaccination with active or weakened probiotic strains offers several advantages over traditional vaccines, such as being noninvasive and nonpathogenic.

The transition to an immune-suppressive tumor microenvironment (TME) has also been blocked as a promising strategy for treating CRC. Researchers have increasingly focused on ginsenosides and their ability to reverse the immune-suppressive TME, especially in the context of their impact on the colorectal TME structure. The study by Qian et al. delves into the immunomodulatory effects of ginsenosides, emphasizing their role and mechanisms in enhancing immune responses in the TME. By incorporating the latest clinical research findings, this study thoroughly assesses the potential and prospects of using ginsenosides to treat CRC, offering a scientific foundation and reference for future clinical applications.

Several studies have indicated that certain microorganisms and their byproducts may influence the development or prevention of tumors through various mechanisms. Recent discoveries have revealed that the gut microbiome and its byproducts can function as either promoters or inhibitors of cancer. Disruption of the expression of noncoding RNAs (ncRNAs) mediated by the gut microbiome has been identified as a cause of gastrointestinal cancer. Ağagündüz et al. provided an overview of the most recent findings on the links among the gut microbiome, ncRNAs, and gastrointestinal cancer. Furthermore, the gut microbiota and its byproducts have a significant effect on the onset of gastric cancer. The importance of microbial treatments such as diet, phages, probiotics, and fecal microbiota transplantation in addressing gastric cancer should not be overlooked. Wang et al. investigated the mechanisms involved in the influence of external pathogen infection and internal microbiota imbalance on the development of gastric cancer. Additionally, the application of microbiota therapy in the treatment and prognosis of gastric cancer has been discussed. The presence of the microbiota has a twofold effect on gastric cancer. First, it can contribute to the progression of gastric cancer and negatively influence its treatment and prognosis through pathogenic bacterial infections and dysbiosis. Second, there is a potential for microbial therapies to address gastric cancer by optimizing the microbiota through dietary modifications, employing bacteriophages and probiotics, and even resorting to FMT.

However, after considering the *in vivo* and *in vitro* studies mentioned, further research through clinical trials is essential to fully establish the anticancer properties of probiotics. Extensive human trials are also necessary to verify the effectiveness of this approach. Indeed, it is crucial to carefully plan experiments to assess the positive and negative effects of probiotics. Further studies should focus on conducting more in-depth research to elucidate the molecular mechanisms of newly discovered probiotic strains derived from functional dairy and nondairy food products, thereby bridging the gap between the food and pharmaceutical sectors.
